# A Small Genome amidst the Giants: Evidence of Genome Reduction in a Small Tubulinid Free-Living Amoeba

**DOI:** 10.1093/gbe/evae058

**Published:** 2024-03-20

**Authors:** Yonas I Tekle, Hanna Tefera

**Affiliations:** Department of Biology, Spelman College, 350 Spelman Lane Southwest, Atlanta, GA 30314, USA; Department of Biology, Spelman College, 350 Spelman Lane Southwest, Atlanta, GA 30314, USA

**Keywords:** Amoebozoa, C-value paradox, evolution, horizontal gene transfer, genome size, Tubulinea

## Abstract

This study investigates the genomic characteristics of *Echinamoeba silvestris*, a small-sized amoeba within the Tubulinea clade of the Amoebozoa supergroup. Despite Tubulinea’s significance in various fields, genomic data for this clade have been scarce. *E. silvestris* presents the smallest free-living amoeba genome within Tubulinea and Amoebozoa to date. Comparative analysis reveals intriguing parallels with parasitic lineages in terms of genome size and predicted gene numbers, emphasizing the need to understand the consequences of reduced genomes in free-living amoebae. Functional categorization of predicted genes in *E. silvestris* shows similar percentages of ortholog groups to other amoebae in various categories, but a distinctive feature is the extensive gene contraction in orphan (ORFan) genes and those involved in biological processes. Notably, among the few genes that underwent expansion, none are related to cellular components, suggesting adaptive processes that streamline biological processes and cellular components for efficiency and energy conservation. Additionally, our investigation into noncoding and repetitive elements sheds light on the evolution of genome size in amoebae, with *E. silvestris* distinguished by low percentage of repetitive elements. Furthermore, the analysis reveals that *E. silvestris* has the lowest mean number of introns per gene among the species studied, providing further support for its observed compact genome. Overall, this research underscores the diversity within Tubulinea, highlights knowledge gaps in Amoebozoa genomics, and positions *E. silvestris* as a valuable addition to genomic data sets, prompting further exploration of complexities in Amoebozoa diversity and genome evolution.

**Significance**

This study addresses a gap in our understanding of amoebae genomics by focusing on *Echinamoeba silvestris*, a small-sized amoeba within Tubulinea. While conventional expectations suggest genome reduction in parasites, our findings reveal intriguing parallels between the free-living amoeba *E. silvestris* and parasitic amoebae lineages in terms of genome size and predicted gene numbers. The study not only sheds light on the evolutionary significance of genome size in free-living amoebae but also contributes valuable insights into the unique genomic features of *E. silvestris*. By exploring the correlation between genome size and cellular characteristics, this research adds complexity to our understanding of amoebae genomics and eukaryotic genome evolution.

## Introduction

The supergroup Amoebozoa is composed of microbial eukaryotes of predominantly amoeboid protists (Cavalier-Smith et al. 2004; Tekle et al. 2008; Smirnov et al. 2011). It encompasses three major lineages including Discosea, Tubulinea, and Evosea (Tekle et al. 2022b). Among this, the Tubulinea is the most robust clade recovered in most molecular phylogenetic studies and most of its members share morphological recognizable tubular pseudopodia (Smirnov et al. 2011; Lahr et al. 2013). Tubulinea encompasses a variety of groups, with notable representatives such as testate amoebae. This adds to its paleontological significance as microorganisms with a protective shell or test. These amoebae play a vital role in the dating of microfossils and the comprehension of historical ecological conditions, serving as bioindicators (Porter et al. 2003; Freitas et al. 2022). Other examples include the classic textbook amoeba genus (order Euamoebida), like *Amoeba proteus*, with members presumed to possess the largest genome all living things (Friz 1968; Porter et al. 2003; Smirnov et al. 2011). Moreover, Tubulinea includes some newly added obscure amoebae members such as *Trichosphaerium* considered to have alternation of generation (Schaudinn 1899; Sheehan and Banner 1973) as well as small-sized amoeba commonly found in human environments such as hospitals causing health concern to humans (Delafont et al. 2019). Despite the familiarity and the health, ecological, and paleontological importance, genome data representing this clade are scarce.

Genome data in the Amoebozoa is steadily growing although majority of the available genomes is from restricted genera that include model organisms or those implicated in human health (Eichinger et al. 2005; Loftus et al. 2005; Clarke et al. 2013). Most genome data that are publicly available for the Amoebozoa belong to the clade Evosea (24 total of these 12 are annotated) and Discosea (18 total, only 2 annotated genomes) (Chelkha et al. 2020; Tekle et al. 2022a, 2023; Zahonova et al. 2022). Tubulinea is only represented by two genomes including *Vermamoeba* (Chelkha et al. 2020) and the recently published *Trichosphaerium* genome (Tekle et al. 2023). The majority of published Amoebozoa genomes exhibits relatively small sizes, averaging around 43 megabase pairs (MB), which is notably smaller compared with the reported genome sizes for this group. A Tubulinea member, *Amoeba dubia*, is purported to possess the largest genome size among all known living organisms, exceeding the human genome by 200 times (Friz 1968). It is important to note that this observation relies solely on qualitative data and necessitates additional confirmation. For instance, numerous amoebae undergo life cycles involving a polyploid stage (Tekle et al. 2014; Goodkov et al. 2020), emphasizing the need to complement qualitative data with sequence or quantitative measurements for accuracy. In this study, we present the draft genome of an isolate belonging to a clade comprising small tubulinid amoebae characterized by special adaptation and posing potential health concern to human.

*Echinamoeba* is a genus of flat, small amoebae that lack protective coverings, are single-nucleated, and exhibit distinctive, spinelike subpseudopodia (Page 1967; Page 1975; Rohr et al. 1998; Baumgartner et al. 2003). Species belonging to this genus are described from thermal and nonthermal natural environments, as well as from hospital hot water systems. The closest relative of the genus is *Vermamoeba vermiformis*, within the order Echinamoebida, and is known for its inhabitance in human environments and pathogenicity (Page 1967; Page 1975; Rohr et al. 1998). Members of Echinamoebida are groups of amoebae adapted to extreme environments, and their potential impact on human health is a concern (Fields et al. 1989; Baumgartner et al. 2003). *V. vermiformis* is the only and the first genome sequenced for the clades Echinamoebida and Tubulinea, respectively (Chelkha et al. 2020). This study aims to address significant knowledge gaps in genomics of the Amoebozoa supergroup, specifically focusing on the Tubulinea clade. The research centers on the small amoeba *Echinamoeba silvestris* CCAP 1519/1 to analyze its genome, aiming to reveal insights into its evolution, adaptation, and potential pathogenicity.

## Results

### Genome Architecture and Gene Prediction of *E. silvestris CCAP 1519/1*

Using 3 sequencing technologies, we generated over 161.4 million sequencing reads (>1,000× coverage), comprising 151.3 million Illumina short reads (11.63 Gb) and long reads totaling 10.1 (3.26 Gb) and 9.0 (16.68 Gb) million, respectively, from Oxford Nanopore and PacBio. These genomic data were derived from amplified DNA nuclei pellets. To ensure data integrity, we implemented a series of bioinformatics and manual curation steps described in the methods to eliminate contamination from known food bacteria or associated entities, symbionts (e.g. viruses, archaea), and other environmental contaminants. From these decontaminated data, we successfully generated a draft genome of *E. silvestris*, totaling 27.1 MB (Table 1). The assembly comprises a total of 284 scaffolds, with an average scaffold length of 95,403 base pairs (bps). *E. silvestris*'s genome falls within the higher GC content range (47.77%) among amoebozoan genomes (Tables 1 and 2).

**Table 1:** Genomic composition and gene model repertoire of *E. silvestris* CCAP 1519/1

Feature	Composition
Genome size (bp)	27.1 MB
GC content (%)	47.77
DNA scaffolds	284
Longest scaffold length (bp)	3,419,598
Shortest scaffold length (bp)	1,192
Mean scaffold length (bp)	95,403
N50 (bp)/L50	984,026
Total number of predicted transcripts	8,327
Proportion of transcripts with a size ≧ 300 bp	6,034
Genes assigned to COGs	8,327
Non-ORFan genes	7,017
ORFan genes	1,310
Mean number of introns/gene	0.9
Mean number of exons/gene	1.9
Mean intron size (bp)	164.7
Mean exon size (bp)	842.1
Gene model BUSCO completeness (complete + partial)	83.2%

**Table 2:** Genomic comparison of diverse Amoebozoan genomes

Species	Genome size—MB	(%) GC content	Predicted genes (CDs)	ORFan genes	Repeat elements	Mean no. introns/gene	Mean no. exons/gene
*E. silvestris*	27.1	47.77	8,327	155	6.83	0.9	1.9
*V. vermiformis*	59.55	41.7	22,473	7,220	9.62	2.7	3.6
*Trichosphaerium* **sp.**	70.8	37.7	27,369	7,489	14.54	8.7	7.7
*E. histolytica*	20.15	24.3	8,163	2	30.78	NA	0.77
*D. discoideum*	34.21	22.5	13,315	63	34.34	NA	0.46
*C. minus*	50.5	26.33	19,925	5,723	12.84	4.7	5.7
*A. castellanii*	42	57.9	14,969	0	4.04	6.2	7.1

The overall genomic content of *E. silvestris* including introns, exon, and gene numbers is smaller compared with published free-living amoebozoan genomes (Eichinger et al. 2005; Loftus et al. 2005; Clarke et al. 2013). The prediction of gene models of *E. silvestris* was aided by transcriptome data from a closest relative *Echinamoeba exundans* (Kang et al. 2017) and a published genome of an amoeba, *Acanthamoeba castellanii* (Clarke et al. 2013). Using this approach, we generated a total of 8,327 gene models. Almost all transcripts obtained from the *E. exundans* transcriptome were found in the draft genome of *E. silvestris* with high similarity percentage matches. In addition to this, the majority, 84.3% (7,017), of gene models was assigned to well-known biological processes in the Clusters of Orthologous Groups of proteins (COGs) database. These include the cellular processes and signaling (27.8%), metabolism (21.3%), information, storage, and processing (17.9%) categories (supplementary table S1, Supplementary Material online). The remaining 20.9% COG category included genes that are poorly characterized or of unknown function (see supplementary table S1, Supplementary Material online). Despite its small genome, *E. silvestris* possesses a similar number of common genes, including ribosomal and cytoskeletal genes (data not shown), as well as genes involved in meiosis (see below), comparable with other amoebae.

The *E. silvestris* draft genome comprises a very small fraction of ORFans (1.86%; Table 1). ORFans are genes that lack BLAST hits in the National Center for Biotechnology Information (NCBI) GenBank database and likely represent novel or unique genes specific to the amoeba. While the exact functions of ORFans remain unknown, some of these genes are expressed, as evidenced by their presence in the *E. exundans* transcriptome data. A preliminary functional exploration of some of these genes, using InterPro, has unveiled some common protein domains in some of these ORFans. These domains include those involved in protein–protein interactions (e.g. lucine-rich and ankyrin repeats) and membrane-associated proteins (e.g. GRAM domain).

### Taxonomic Distribution and Interdomain LGT of Predicted Gene Models in *E. silvestris*

**Fig. 1.**
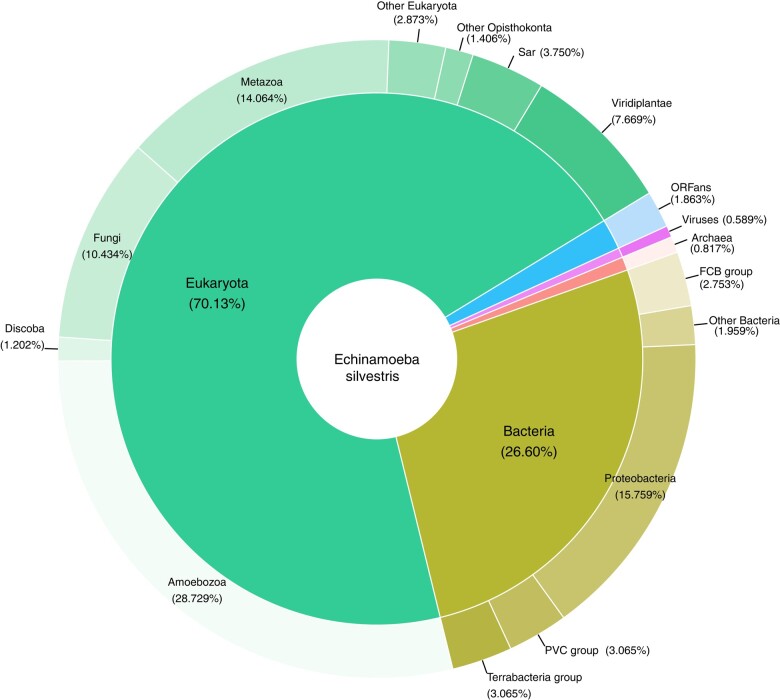
Taxonomic classification of predicted proteins deduced from *E. silvestris* draft genome.

Similar to other amoebae genomes, the *E. silvestris* genome displays a diverse distribution of gene models across various taxonomic groups. The majority (70.13%) of the gene models matched eukaryotic genes (Fig. 1). Among other living domains, the largest proportion (26.6%, 2,215 genes) shows highest similarities to bacteria, while only a small fraction shows similarities to archaeal genes (0.8%, 67 genes) (Fig. 1). An even smaller percentage of gene models (0.6%, 49 genes) show similarity to viral genes. This latter set, noneukaryote matching genes, makes up core components of cellular (signaling and metabolism) and information storage and processing (supplementary fig. S1, Supplementary Material online). The expression of some of these gene models with high similarity to bacterial, archaeal, and viruses has been detected in transcriptome data of *E. exundans* (supplementary table S2, Supplementary Material online).

To assess evidence for lateral gene transfer (LGT) of the noneukaryotic predicted gene model, we conducted alien index (AI) analyses and phylogenetic analysis on selected genes (supplementary fig. S2, Supplementary Material online). Based on BLAST similarities and AI analyses, we identified a large number of LGT candidates from bacteria and archaea. The likely donors of the putative LGTs in bacteria come from diverse taxonomic groups but most prominently (66%) from Pseudomonadota (Proteobacteria). Other major potential donors include PVC group (10.7%), FCB group (7.2%), and Terrabacteria group (4.7%) (see supplementary fig. S2a and b and table S2, Supplementary Material online).

Among the 67-archaeal origin genes found in the *E. silvestris* draft genome, only 2 genes had an AI above the threshold (supplementary table S2, Supplementary Material online). A phylogenetic analysis of the putative archaeal LGT showed that both *E. silvestris* and *V. vermiformis* acquired a similar gene that encodes for lactate dehydrogenase-like protein (supplementary fig. S2c, Supplementary Material online). The likely donor of these putative genes is Euryarchaeota.

Similarly, *E. silvestris* have a dozen of putative LGTs, with AIs above the threshold, of viral origin (supplementary fig. S2d and table S2, Supplementary Material online). Majority of these putative LGTs has their origins from giant virus clade Mimiviridae, which include *Acanthamoeba polyphaga* mimivirus, Klosneuvirus KNV1, *Hyperionvirus* sp., and Fadolivirus. The others unclassified giant viruses including Kaumoebavirus and Pithovirus (supplementary table S2, Supplementary Material online). The former giant virus has been reported to be associated with a closest relative of *V. vermiformis* (Bajrai et al. 2016).

### Comparative Genomics and Genes Involved in Sexual Life Cycle

**Fig. 2.**
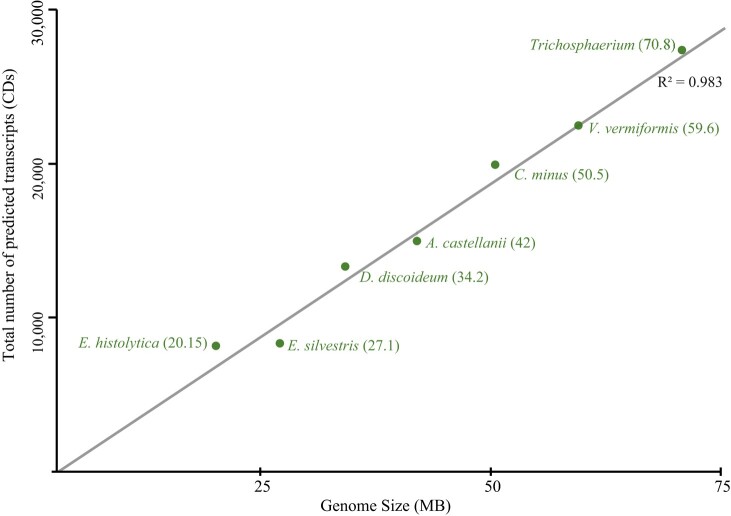
Correlation between genome size and number of predicted genes in Amoebozoans.

Comparative genome analysis of amoebae representing the three clades of amoebae, Tubulinea (*E. silvestris*, *V. vermiformis*, and *Trichosphaerium* sp.), Discosea (*A. castellanii* and *Cochliopodium minus*), and Evosea (*Entamoeba histolytica* and *Dictyostelium discoideum*), unveils intriguing findings, as detailed in Table 2. *E. silvestris* is the smallest free-living genome to the exclusion of the parasitic *E. histolytica*. Notably, *E. silvestris* exhibits a substantially lower number of predicted gene models comparable with the parasitic amoebae (see Table 2). Despite variations in morphological size, most sequenced free-living amoebae genomes consistently exhibit an average of ∼17,500 genes (ranging from 13,315 to 27,369). The analysis of genome size versus the number of predicted gene models reveals robust correlations (Fig. 2).

A detailed examination of the genome architecture reveals that *E. silvestris* has the lowest average number of introns per gene compared with its closest relatives in the Tubulinea clade and other members of Discosea (Table 2). Intron information for members of Evosea is not available. Analyzing the number of ORFan genes among Tubulinea members, *E. silvestris* emerges with the lowest total percentage. However, a cross-amoebae comparison of ORFan genes proves challenging due to the comprehensive classification of genes in model and parasitic organisms resulting from extensive studies. Nevertheless, it is noteworthy that among Tubulinea members whose genomes are assembled and annotated with a similar approach, *E. silvestris* is shown to have a significantly lower number of ORFan genes (Table 2). Similarly, *E. silvestris* exhibits the lowest percentage of repetitive elements in its genome (Table 2).

The analyzed amoebae display a range of GC content in their genomes. In general, members of Tubulinea exhibit higher average GC content, while members of Discosea and Evosea have lower GC content, with one exception: *A. castellanii*, which has the highest GC content among all studied amoebozoans so far (Table 2).

Comparative genomics also reveals that *E. silvestris* has experienced extensive gene family contraction in comparison with other amoebae (supplementary fig. S3 and table S2, Supplementary Material online). Similarly, the number of gene families undergoing expansion is notably lower in *E. silvestris* (supplementary fig. S3, Supplementary Material online). The majority of gene families undergoing expansion and contraction is associated with biological processes and molecular functions, with only a few classified under cellular components (see supplementary table S3, Supplementary Material online). Among the expanded gene families are those involved in nuclear import, cell movement, and division, as well as proteins contributing to apoptosis and pathogen defense (supplementary table S3, Supplementary Material online).

To explore the sexuality of *E. silvestris* and assess genome completeness using well-known genes in amoebae genomes, we conducted a gene inventory analysis of meiosis-specific genes (supplementary table S4, Supplementary Material online). *E. silvestris* possesses almost all known meiosis-specific genes in amoebae and other eukaryotes (supplementary table S4, Supplementary Material online). Two genes, *MND1* and *HOP2*, were not found in this draft genome. Although the absence of these genes might be due to the incompleteness of the draft genome, it is common to observe the absence of one or a few meiosis genes in several amoebae genomes, including confirmed cases such as the absence of *SPO11* and *DMC1* in *D. discoideum* (supplementary table S4, Supplementary Material online).

## Discussion

### *E. silvestris*: the Smallest Free-Living Amoebae Genome to Date

Comparative genomics of Amoebozoans, encompassing understudied groups, is yielding valuable insights into the evolution and genetic diversity within this group (Chelkha et al. 2020; Tekle et al. 2021, 2022a, 2022b, 2023; Zahonova et al. 2022). Until now, our comprehension of Amoebozoa supergroup genomes has been constrained by well-studied model organisms and other traditional (qualitative) genome data resources, often lacking in-depth details (Eichinger et al. 2005; Loftus et al. 2005; Glockner and Noegel 2013). Here, we present a draft genome of an amoeba belonging to a group comprised of small-sized amoebae, encompassing lineages with potential pathogenicity and adaptation to extreme temperatures (Baumgartner et al. 2003; Delafont et al. 2018). This study contributes a third genome data set to the Tubulinea clade, the least examined clade in genomic studies of the Amoebozoa supergroup (Chelkha et al. 2020; Tekle et al. 2023). Tubulinea encompasses diverse lineages of amoebae, exhibiting variations in behavior, morphology, and ecology (Smirnov et al. 2011). Traditionally recognized as a clade possessing large genome sized members (Friz 1968), our study reveals the smallest free-living amoeba genome within the Tubulinea clade. This underscores the substantial diversity within the Tubulinea clade and emphasizes the existing knowledge gaps in Amoebozoa genomics and diversity.

While the available data on the nature and diversity of genomes in Tubulinea is limited, our discovery of the small genome observed in *E. silvestris*, comparable with a parasitic lineage, is intriguing in its own right. Among the analyzed amoebae genomes, the only genomes closest in size and number of predicted gene models are those of the parasitic genus *Entamoeba* (Table 2). The average genome size in free-living amoebae, regardless of morphology or life history complexity, is 43 MB (see Table 2). The genome of *E. silvestris* is approximately half the size of its closest relative, *V. vermiformis*, and about 20% smaller compared with the smallest (before the current study) free-living amoeba, *D. discoideum* (Table 2 and Fig. 2). The consequences of reduced or small genomes and their evolutionary significance in free-living amoebae and other microbial eukaryotes are not well understood due to limited data (Zhang et al. 2022).

A previous report showed that cell size is correlated with genome size across many taxonomic groups (Bennett and Leitch 2005; Gregory 2005). *E. silvestris* (∼11 µm) is the smallest in size compared with the amoebae examined in this study. This observation supports these previous reports; however, cell sizes in the studied amoebae have a close range (ranging 10 to 60 µm), with some of them only a few microns apart and still displaying large proportions of genomic size variations among cells of similar size. Therefore, more data are needed to examine the correlation between cell and genome size in amoebae. It is worth noting that the division (growth) rate has been observed to correlate with genome size, and the trend suggests that organisms with smaller genomes tend to exhibit higher division rates compared with those with larger genomes (Bennett and Leitch 2005; Gregory 2005). Despite possessing the smallest genome, *E. silvestris* stands out as one of the slowest-dividing amoebae among the free-living amoebae we cultivate in our laboratories under similar conditions. This adds complexity to the association between genome size and cellular characteristics. The association between genome size and organismal complexity is one of the contentions and complex subjects in eukaryotic evolution. This is generally called the C-value enigma and is mostly based on observations obtained in macrobial (animals and plants) organisms (Thomas 1971). The C-value enigma refers to the perplexing observation that the amount of DNA (measured in terms of C-value, representing the DNA content) in an organism’s genome does not necessarily correlate with its complexity or perceived biological complexity. That is, organisms with more complex structures or functions do not always have larger genomes. This phenomenon challenges the intuitive expectation that more advanced or intricate organisms should have larger amounts of genetic material. The C-value enigma has prompted scientific inquiry into understanding the nonlinear relationship between genome size and organismal complexity. With more genome data from microbial eukaryotes, it is possible to gain insights into this intriguing topic, considering factors such as body (cell) size, metabolism, developmental rate, and geographical distribution.

### Genome Architectural Evidence for Small Genome Size in *E. silvestris*

The intricacies of genome evolution, particularly concerning size, in eukaryotes are multifaceted. Eukaryotic genomes exhibit a substantial size range compared with prokaryotes, a phenomenon influenced by various additional genomic features and evolutionary forces shaping their development. Determinants of genome size in eukaryotes include factors such as functional complexity (gene content), repetitive sequences, transposable elements, ploidy level, noncoding DNA, and introns (Elliott and Gregory 2015). Additionally, the evolutionary history, marked by events such as duplications, deletions, and rearrangements, along with environmental factors, serves as additional determinants influencing the evolution of genome size in eukaryotes. In this study, we conducted a comparative genomics analysis on the draft genome of *E. silvestris* to elucidate specific aspects related to the evolution of genome size in amoebae.

Our findings provide multiple lines of evidence supporting the observed small genome size in *E. silvestris*. Gene content emerges as a prominent contributor to genome size in amoebozoans (Table 2 (Eichinger et al. 2005; Delafont et al. 2018; Tekle et al. 2022a, 2023)) and other microbial eukaryotes (Elliott and Gregory 2015). The total predicted number of genes in amoebae exhibits a robust correlation with genome size (Fig. 2). Notably, *E. silvestris* has the lowest number of predicted genes among the free-living amoebae examined in our study (Table 2). While limited publicly available genome data exist for amoebae (Clarke et al. 2013; Tekle et al. 2022a; Zahonova et al. 2022), our preliminary data on other diverse amoebae with comparable size appear to have substantially more predicted genes than *E. silvestris* (Tekle, unpublished data).

Surprisingly, the number of predicted genes in *E. silvestris* is comparable with that of parasitic amoebae (Table 2). This observation is intriguing considering that parasitic lineages are generally considered to have reduced genomes due to adaptive processes associated with optimizing the parasitic lifestyle (Keeling and Slamovits 2005; Corradi and Slamovits 2011; Jackson 2015). Such processes include evolutionary pressures favoring the loss of nonessential genes and pathways, driven by factors such as host dependency, energy efficiency, symbiosis, specialization, and reliance on host functions. Parasitic lineages of amoebae, exemplified by *E. histolytica*, also lack mitochondria, further impacting their overall functional genetic composition.

Functional categorization of predicted genes in *E. silvestris* reveals similar percentages of genes assigned to cellular processes and signaling, metabolism, information storage, and processing, as observed in other amoebae (supplementary table S1, Supplementary Material online). *E. silvestris* also have a similar number of essential genes such as cytoskeleton and ribosomal as well as those genes involved in meiosis (supplementary table S4, Supplementary Material online). However, *E. silvestris* appears to have undergone extensive gene contraction relative to other amoebae (supplementary fig. S3 and table S3, Supplementary Material online). Two noteworthy observations include the substantial number of gene contractions in genes involved in biological processes, and among the few genes that underwent expansion, none are related to cellular components (supplementary table S3, Supplementary Material online). It is plausible that the small size of the amoeba has led to adaptive processes streamlining biological processes and cellular components for efficiency and energy conservation.

In our investigation of gene content within less-explored amoebae genomes, a noteworthy observation emerges from the substantial presence of genes lacking significant homology in public databases, commonly known as ORFans. These ORFans constitute a considerable proportion of amoebae genomes and likely contribute to shaping unique morphological features and other adaptations specific to amoebae (Tekle et al. 2022a). Notably, *E. silvestris* has a significantly lower percentage of ORFans (4.7%) compared with its closely related species (*V. vermiformis*, 32%, and *Trichosphaerium* sp., 27%) and a distantly related species, *C. minus* (29%), potentially accounting for its smaller genome size. However, it is crucial to interpret these findings with caution, considering that extensively studied and well-characterized genomes, such as those of *E*. *histolytica*, *D. discoideum*, and *A. castellanii*, exhibit minimal numbers of ORFans. Consequently, while our comparative analysis suggests a significant reduction of ORFans in the *E. silvestris* genome compared with its close relatives, whose genome data are collected using a similar approach, further validation is essential as our comprehension deepens with improved amoebae genome characterization. The functional characterization of ORFans, representing a substantial portion of the genome, holds the promise of unraveling intriguing insights into the evolution and diversification of this group.

Our comparative analysis of noncoding and repetitive elements provides additional insights into the genome size evolution of amoebae. Noncoding DNA constitutes a significant contributor to genome size evolution in eukaryotes, encompassing various elements such as promoters, enhancers, silencers, noncoding RNA molecules, intergenic regions, introns, and repetitive elements. While the nature and contribution of noncoding DNA in multicellular eukaryotes are well documented (Elliott and Gregory 2015), variations may exist across species or taxonomic groups. On average, noncoding DNA constitutes 27% and 51% of the genome in animals and plants, respectively (Elliott and Gregory 2015). In some cases, such as in mammals and other vertebrates, the majority of their genome (>90%) is composed of these elements (Mattick and Makunin 2006; de Koning et al. 2011). The nature and evolution of noncoding regions in genomes of microbial eukaryotes in general, and amoebae in particular, are poorly understood (Elliott and Gregory 2015). Limited studies on repetitive elements, focusing on the parasitic lineages of amoebae, exist, reporting varying types of repetitive elements (Bakre et al. 2005; Lorenzi et al. 2008). In this study, we compiled a large database of repetitive elements and conducted a comprehensive comparison within amoebae representing the three major groups. We found consistent results with various types of noncoding elements in amoebae. A detailed report of this study will be published elsewhere. Here, we report the contribution of repetitive elements to genome size in relation to *E. silvestris*. Among the species analyzed, *E. silvestris* has the lowest percentage of repetitive elements in its genome. Members of Evosea (*E. histolytica* and *D. discoideum*) have the highest percentages of repetitive elements (Table 2). While a more thorough study is required to investigate the variation observed between the major clades, this result clearly demonstrates that repetitive elements significantly contribute to the genome size evolution of amoebas, similar to multicellular organisms (Elliott and Gregory 2015). The lower percentage of repetitive elements in *E. silvestris* corroborates their contribution to genome size. Repetitive elements are found to positively correlate with genome size in other eukaryotes (Elliott and Gregory 2015), although such a conclusion cannot be reached in amoebae due to limited sampling. Similarly, *E. silvestris* has the lowest mean number of introns per gene, further supporting the observed reduced genome in this amoeba. A similar pattern of genome reduction in repeat elements and other noncoding regions is also known in one of the smallest free-living eukaryotes, green alga, *Ostreococcus tauri* (Derelle et al. 2006).

## Conclusion

In conclusion, our study presents the draft genome of *E. silvestris*, the smallest free-living amoeba genome identified to date. This research contributes to the comparative genomics of amoebozoans, shedding light on the evolution and genetic diversity within this group. The exploration of the Tubulinea clade, one of the least sampled clades in amoebozoan genomics, underscores the significant diversity within this lineage. Despite the limited data on Tubulinea genomes, our findings reveal the unique nature of *E. silvestris*, exhibiting a genome size comparable with parasitic lineages, introducing added complexity to conventional expectations of genome reduction in parasites. Additionally, our investigation into noncoding and repetitive elements provides insights into the evolution of genome size in amoebae, with *E. silvestris* standing out for its low percentage of repetitive elements. However, the observed correlation between genome size and cellular characteristics, such as division rate, adds complexity to the understanding of the evolutionary significance of genome size in free-living amoebae. Further research is warranted to explore the implications of these findings on amoebae genomics and the broader context of eukaryotic genome evolution.

## Materials and Methods

### Genomic DNA Collection of *E.* silvestris CCAP 1519/1

Culture of *E. silvestris* was obtained from Culture Collection of Algae and Protozoa (CCAP). Subsequent monoculture of this isolate was grown for genomic DNA isolation in plastic petri dishes with bottled spring water (Deer Park; Nestlé Corp. Glendale, CA) at room temperature supplemented with autoclaved grains of rice. The identification of the isolate was confirmed using morphological and genetic analysis against published literature for the isolate. *E. silvestris* on average is 11 µm in size with locomotive morphology of triangular or elongate forms. It displays the characteristic spinelike subpseudopodia of the genus (Page 1975; Baumgartner et al. 2003).

Genomic DNA from *E. silvestris* was extracted from monoclonal cultures following a previously published protocol for nuclear extraction and genome amplification (Tekle et al. 2022a, 2023). The nuclear extraction process began by lysing cleaned adherent cells to release the nuclei. This was achieved by incubating the cells in 6 mL of lysis buffer, composed of sodium phosphate buffer at pH 7.4, 5 mM MgCl_2_, and 0.1% Triton-X 100, for 2 h. Subsequently, the lysate containing free nuclei was separated by centrifugation at 500 rpm for 10 min at room temperature. The resulting nuclei pellet was resuspended in 0.5 mL of lysis buffer and carefully layered on top of a 12 mL sucrose cushion consisting of 30% sucrose, sodium phosphate buffer at pH 7.4, and 0.5% Triton-X 100. The sucrose cushion played a crucial role in separating the nuclei by capturing small particles (e.g. bacteria) or lightweight lysates (cell parts) during centrifugation. The mixture of lysate and sucrose cushion was then centrifuged at 3,200 rpm for 20 min at room temperature. The pellet obtained from this step was resuspended in 1 mL of lysis buffer and subjected to further centrifugation at 10,000 rpm for 1 min at room temperature. The purified nuclei pellets were carefully collected after removing the supernatant. Whole-genome amplification (WGA) nuclei pellets were carried out using the REPLI-g Advanced DNA Single Cell Kit (QIAGEN; Cat No./ID: 150363), following the manufacturer’s protocol. Finally, the amplified DNA was quantified using the Qubit assay with the dsDNA Broad Range Kit (Life Technologies, Carlsbad, CA, USA).

### Genomic DNA Library Preparation and Sequencing

We applied three different sequencing strategies: Illumina short reads, 10× genomics-linked reads, and Oxford Nanopore long-read sequencing. For Illumina and PacBio sequencing, we sent a nucleus pellet amplified gDNA sample to GENEWIZ (Azenta Life Sciences, South Plainfield, NJ) for library preparation and sequencing as described in Tekle et al. (2022a). Oxford Nanopore technology (ONT) (Oxford Nanopore Technologies Ltd., United Kingdom) was used to generate long-read sequences in our lab with MinION device using the SQK-RAD004 kit. We constructed the library from 400 µg of amplified nuclear or single-cell gDNA, and this library mix was added to the flow cell using the SpotON port.

### De Novo Genome Assembly and Contaminant Removal

We employed a comprehensive suite of bioinformatics pipelines, building upon our prior work, to assemble and annotate the genome while eliminating contamination (Tekle et al. 2022a, 2023). Initially, we assembled Nanopore and PacBio long-read sequences, using the Canu assembler. Subsequently, this assembly underwent careful refinement over three iterations, integrating high-quality Illumina reads using Pilon v1.2. To further enhance the assembly quality, we applied Redundans to remove heterozygous genome segments and eliminate short (<1,000 bp) contigs. This refined assembly served as the basis for contamination assessment (see below). By aligning trimmed Illumina short reads and Canu-corrected long reads to the polished assembly using minimap2, we determined per-contig (scaffold) coverage. Additionally, we used a diamond-blastx search against the NCBI nt database to assign taxonomic annotations to each scaffold. Scaffolds meeting specific criteria, such as noneukaryotic taxonomic hits and coverage < 10.0, were flagged as contaminants and excluded from subsequent analyses. The remaining scaffolds were employed for gene prediction (see below). To further scrutinize contamination, we performed a BLASTP search against the NCBI nonredundant protein database (nr) for predicted gene models. Scaffolds with significant BLASTP hits to potential contaminants, particularly those specially linked to bacteria, archaea, viruses, or nonamoeboid eukaryotes, were carefully examined and removed. The decontaminated scaffolds were analyzed using BUSCO and transcriptome data to confirm that amoeba (eukaryote) genes were preserved.

### Gene Prediction, LGT Analysis, and Identification of Sex-Related Genes

We followed the methodology for gene annotation described in Tekle et al. (2022a) using transcriptome data from a closely related amoebae *E. exundans* and protein sequences from a published genome of a related amoeba, *A. castellanii* (Clarke et al. 2013). Following the prediction of the finalized gene set, we classified the predicted gene models into COG categories using EggNOG-mapper, as implemented in OmicsBox v.2.0.29. Functional annotations of coding gene were obtained from the best BLAST hit with BLASTP (e-value < 1e^−3^) against the NCBI nr database. Genes that had no hits to nr database were classified as ORFans. Genome annotation quality was evaluated by BUSCO using the Eukaryote odb10 database.

To detect potential LGT from the draft genome of *E. silvestris*, we checked extracted genes that matched to bacteria, virus, and archaea based on their nonredundant annotations as LGT candidate for further analyses. We performed BLASTP to search these genes against archaea, bacteria, and virus genomes that are commonly seen in previous BLAST results. We then performed an AI analysis based on the BLASTP results similar to our published methods (Tekle et al. 2022a) to calculate the AI scores. Genes with AI ≥ 45 were considered as putative LTGs. To further corroborate these results, we built phylogenetic trees for selected putative LTGs in IQ-Tree using the automatic model selection option and 1,000 ultrafast bootstrap replicates.

To identify gene models of certain genes important in adaptive behavior (thermal or pathogenicity) and those implicated in sexual reproduction within the draft genome of *E. silvestris*, we performed BLASTP (e-value 1e^−15^) search of respective genes as in Wood et al. (2017). The selected genes were further analyzed using phylogenetic analysis in IQ-Tree as described above to further assess their homology in phylogenetic framework.

### Repetitive Elements, Gene Family, and Ontogeny Analyses

We constructed a comprehensive nonredundant repeat element database encompassing all Amoebozoans, as outlined in references (Šatović et al. 2020). To compile this database, repetitive elements identified by three distinct software packages (LTRharvest, TransposonPSI, RepeatModeler) were combined into a unified library for each species. The outcomes of these analyses were categorized using the Repeat classifier script within the RepeatModeler package (Flynn et al. 2020). These classifications were further consolidated into a master database, eliminating duplicate repeats through VSEARCH (Rognes et al. 2016). This master database served as the foundation for conducting comparative analyses of repeat elements present in Amoebozoa genomes. The final percentage of repetitive elements within each genome was determined by applying RepeatMasker against this nonredundant database.

For general whole-genome comparative analysis of protein-coding genes, including clustering based on orthology and function, we employed OrthoVenn3 (Wang et al. 2015), a web-based tool. Additionally, OrthoVenn3 functionalities were utilized for gene family expansion–contraction history analysis, employing the CAFE5 (Mendes et al. 2021) software.

## Supplementary Material

Supplementary material is available at *Genome Biology and Evolution* online.

## Supplementary Material

evae058_Supplementary_Data

## Data Availability

The genome data (BioSample accession: SAMN38518977) and other supporting materials are accessible within the article and online as supplementary information.
