# Supplementary material for: A Small Genome amidst the Giants: Evidence of Genome Reduction in a Small Tubulinid Free-Living Amoeba
Source: Genome Biol Evol. 2024 Mar 20;16(3):evae058. doi: 10.1093/gbe/evae058 (PMC10980511; doi:10.1093/gbe/evae058)
Supplement: evae058_Supplementary_Data [file evae058_supplementary_data.zip › Supp Legends.docx]

**Supporting information legends**

**Fig. S1**. Functional categorization of putative HGTs (LGTs) found in *Echinamoeba silvestris* draft genome based on the Cluster Orthologous Groups (COGs) database.

**Fig. S2**. Phylogenetic reconstructions demonstrating putative lateral gene transfers (LGTs) in genome among bacteria (A, B), archaea (C), and giant viruses (D) from *Echinamoeba silvestris*. Clade supports at nodes are ML IQ-TREE 1000 ultrafast bootstrap values. All branches are drawn to scale.

**Fig. S3**. Gene family expansion and contraction based on whole genome coding proteins of amoebae constructed using inbuild function of OrthoVenn3 (Wang, et al. 2015). Pie charts at internodes (shared) and terminal taxon (unique) show number of genes that underwent contraction (negative values - blue) and expansion (positive values - purple).

**Table S1**. Distribution of the gene models of *Echinamoeba silvestris* draft genome in categories of clusters of orthologous groups of proteins (COGs).

**Table S2**. Putative LGT-derived genes in *Echinamoeba silvestris* draft genome with Alien Index above threshold (>45) scores.

**Table S3**. Functional classification of gene families that underwent contraction (sheet1) and expansion (sheet2).

**Table S4**. Meiosis genes inventory in members of Amoebozoa.
