# Supplementary figures and images for: A Small Genome amidst the Giants: Evidence of Genome Reduction in a Small Tubulinid Free-Living Amoeba
Source: Genome Biol Evol. 2024 Mar 20;16(3):evae058. doi: 10.1093/gbe/evae058 (PMC10980511; doi:10.1093/gbe/evae058)

## COG categories of candidate LGTs

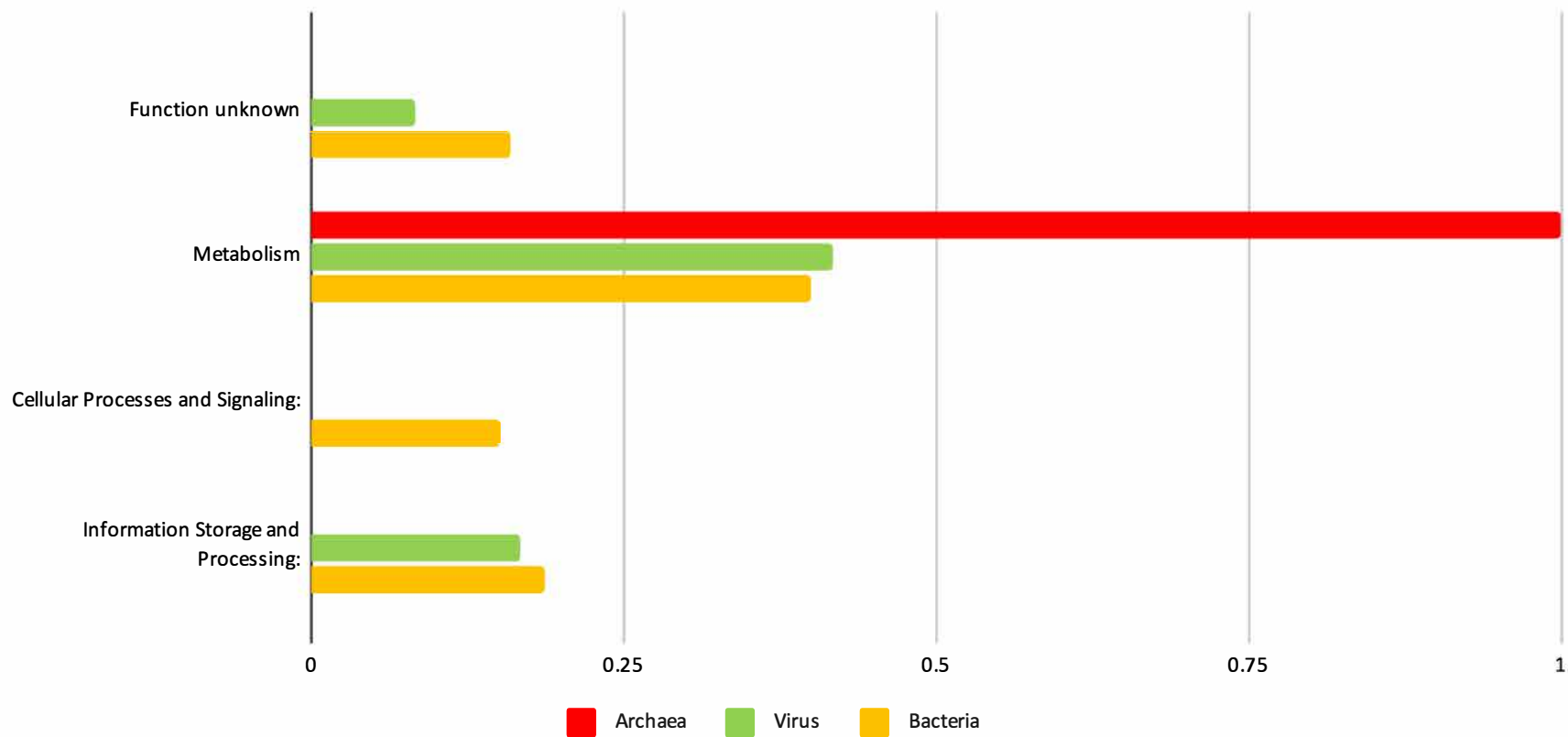

Supplement: evae058_Supplementary_Data [file evae058_supplementary_data.zip › Figure_1S.pdf]

A.

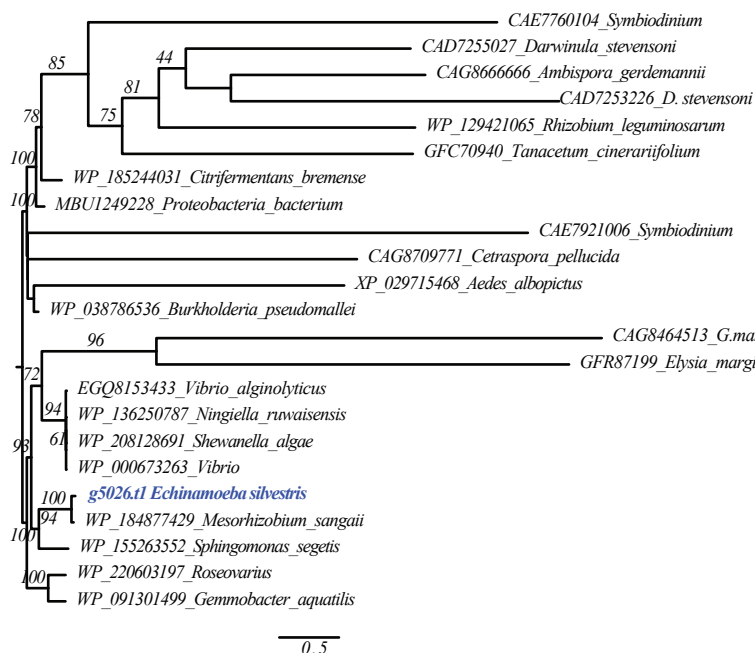

B.

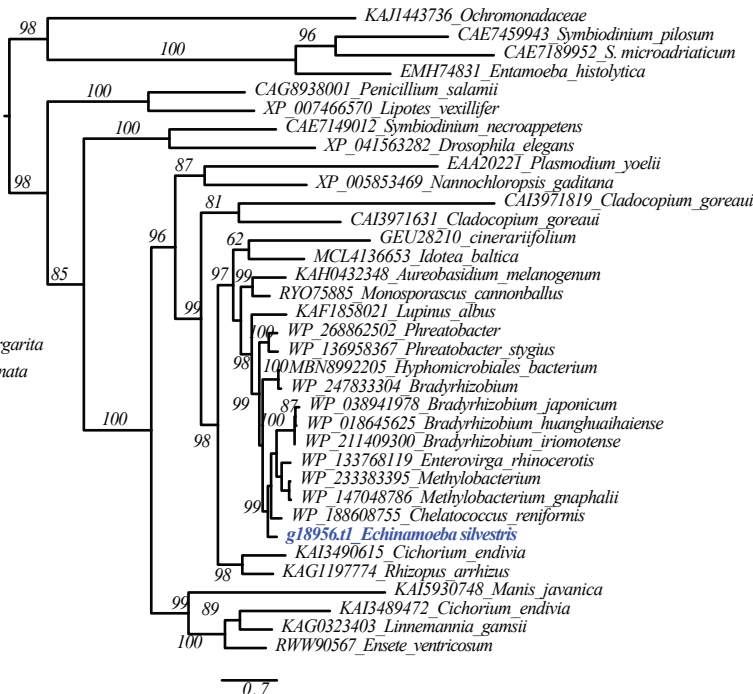

C.

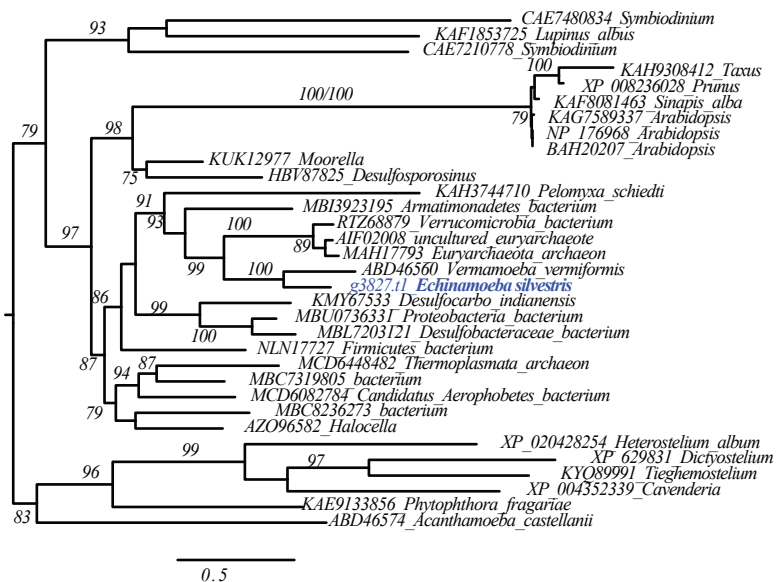

D.

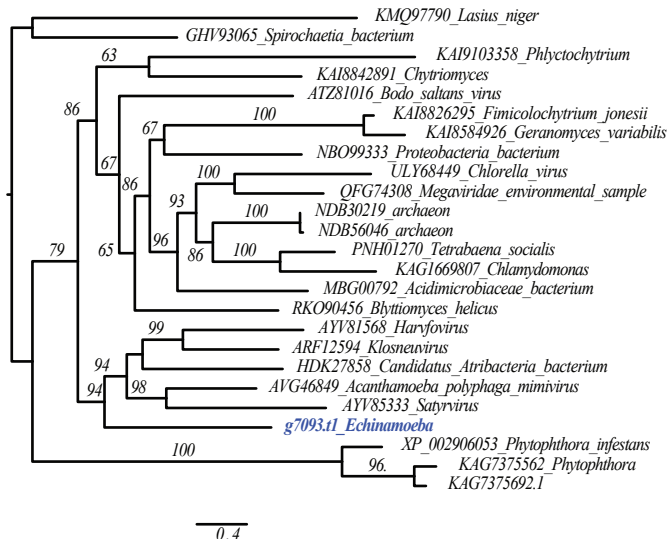

Supplement: evae058_Supplementary_Data [file evae058_supplementary_data.zip › Figure_S2.pdf]

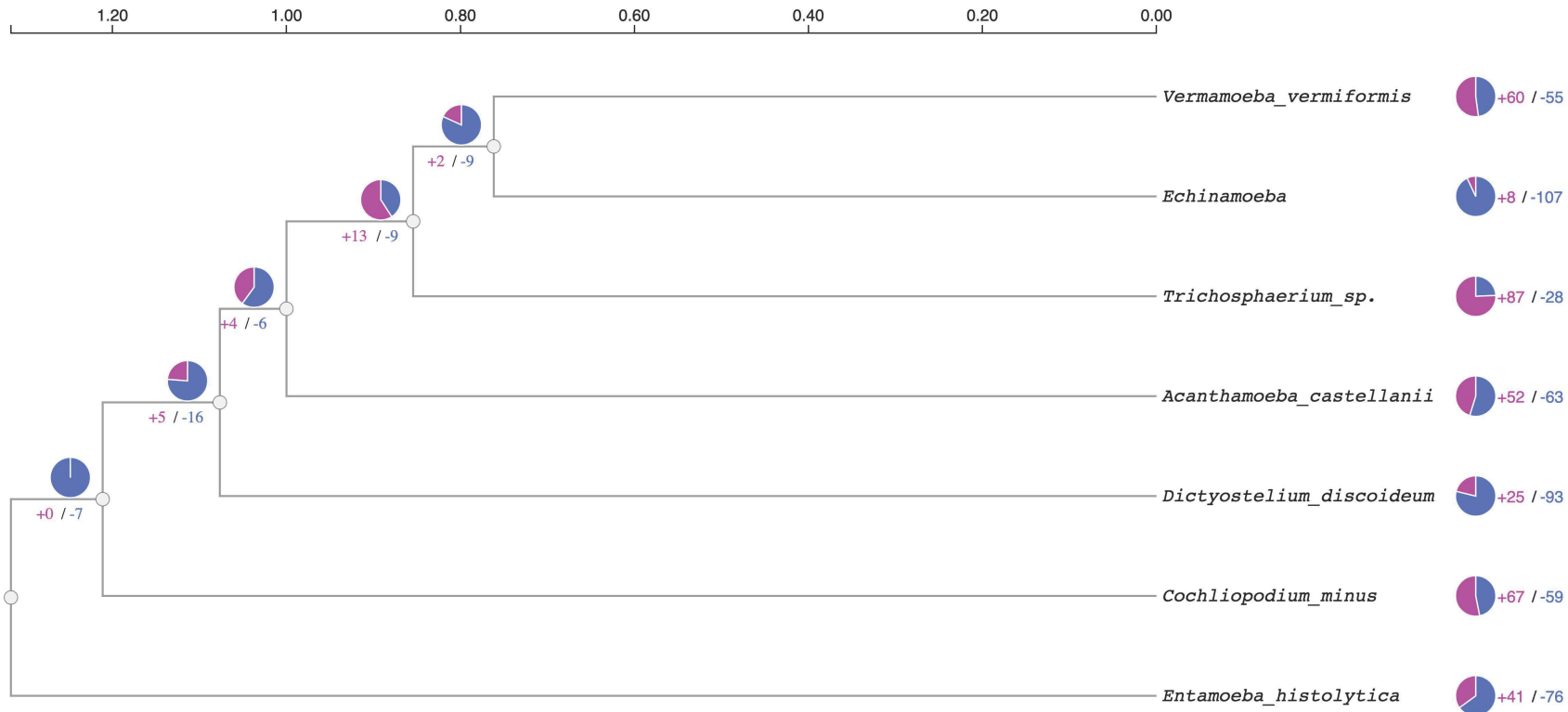

Supplement: evae058_Supplementary_Data [file evae058_supplementary_data.zip › Figure_S3.pdf]

Figure S1

# COG categories of candidate LGTs

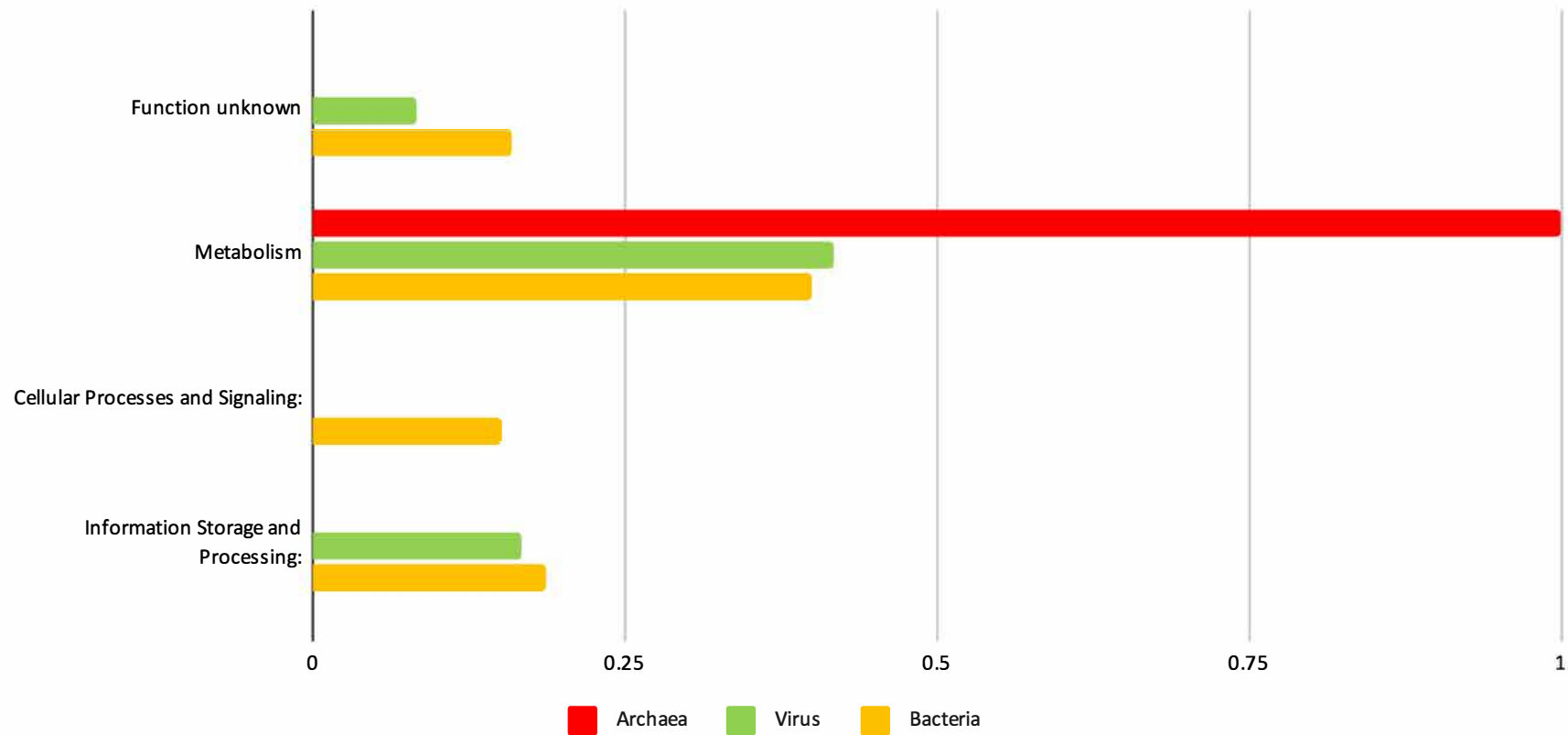

Figure S2

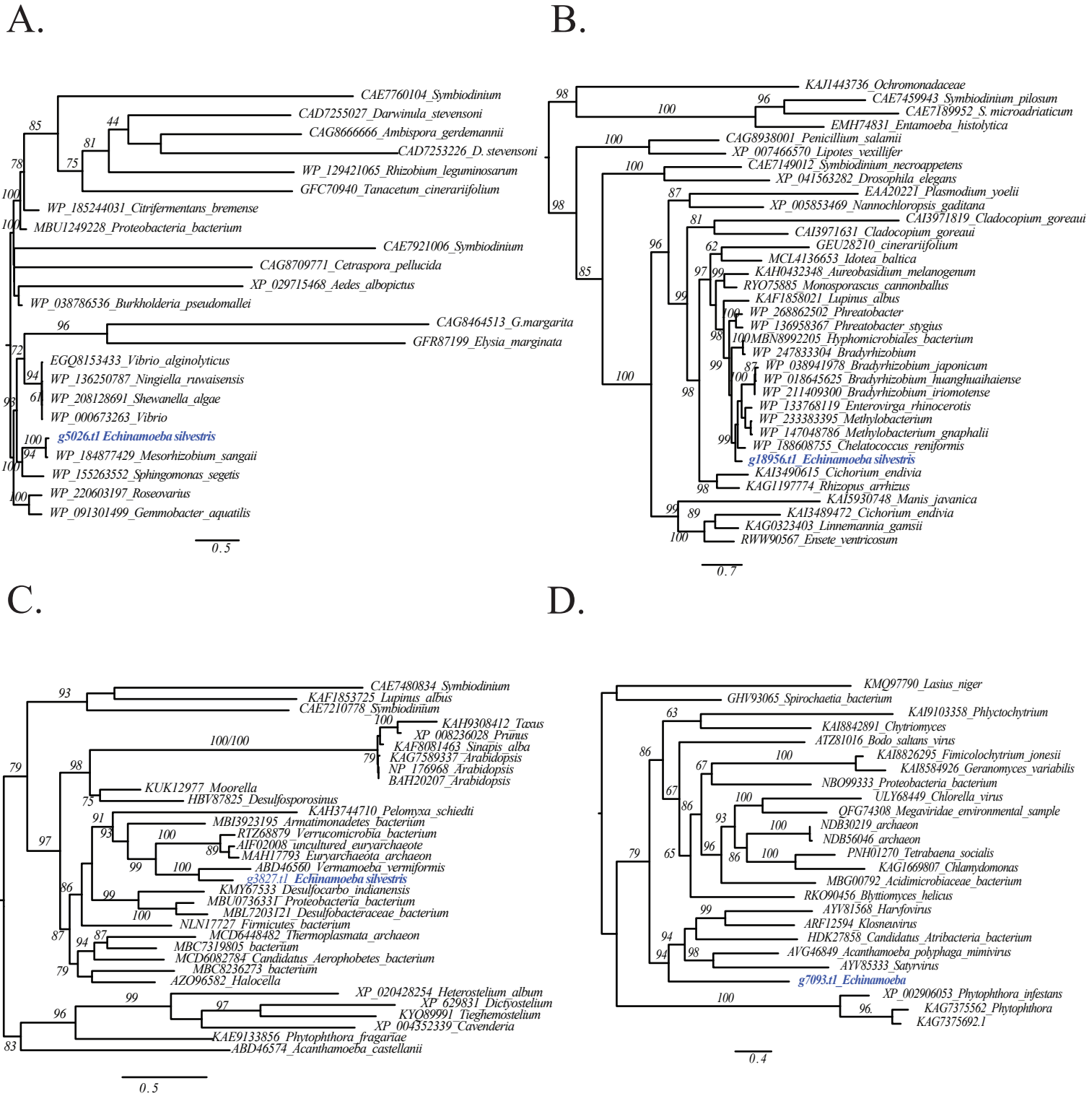

Figure S3

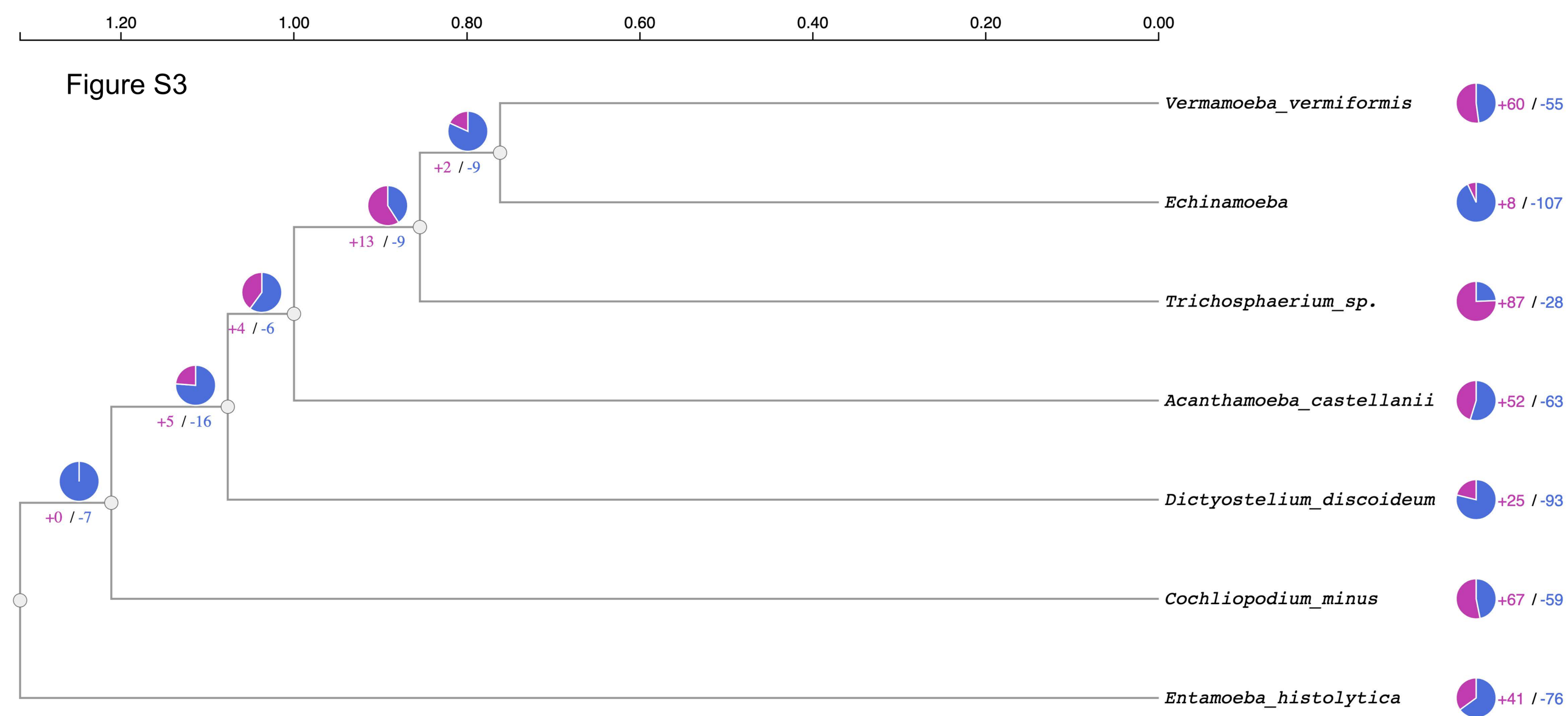

Supplement: evae058_Supplementary_Data [file evae058_supplementary_data.zip › Fig_1S_S3.pdf]
